# Comparative Evaluation of Three Immunoassays for the Simultaneous Detection of *Clostridioides difficile* Glutamate Dehydrogenase and Toxin A/B

**DOI:** 10.3390/microorganisms10050947

**Published:** 2022-04-30

**Authors:** Namsu Kim, Seung Yeob Lee, Joonhong Park, Jaehyeon Lee

**Affiliations:** 1 Department of Laboratory Medicine, Jeonbuk National University Medical School and Hospital, Jeonju 54907, Korea; namsu1224@naver.com (N.K.); seungyeoblee@jbnu.ac.kr (S.Y.L.); 2 Research Institute of Clinical Medicine of Jeonbuk National University-Biomedical Research Institute of Jeonbuk National University Hospital, Jeonju 54907, Korea

**Keywords:** *Clostridioides difficile*, glutamate dehydrogenase, CD toxin A/B, VIDAS *C. difficile* GDH, VIDAS CDAB, RIDASCREEN *C. difficile* GDH, RIDASCREEN *C. difficile* toxin A/B, C. DIFF QUIK CHEK COMPLETE

## Abstract

Background: In the medical laboratory, a step-by-step workflow for *Clostridioides difficile* infection (CDI) detection using glutamate dehydrogenase (GDH) and toxin A/B assays for initial screening, along with a nucleic acid amplification test (NAAT), has been recommended recently. In this study, we evaluated these three immunoassays for the simultaneous detection of GDH and *Clostridioides difficile* (CD) toxin A/B. Methods: A total of 304 stool samples were tested for the presence of GDH antigen and CD toxin A/B using VIDAS *C. difficile* GDH and toxin A/B (CDAB), RIDASCREEN *C. difficile* GDH and toxin A/B (RIDA), and C. DIFF QUIK CHEK COMPLETE according to the manufacturers’ recommendations. As complementary reference methods for GDH and toxin A/B detection in the three immunoassays, CD cultures using ChromID *C. difficile* agar and the Xpert *C. difficile* assay, respectively, were tested. Results: All three GDH assays showed overall substantial agreement with the CD culture. All three toxin A/B assays showed overall moderate agreement with the Xpert *C. difficile* assay. In comparison with consensus results, VIDAS GDH and QCC GDH showed almost perfect agreement, whereas RIDA GDH showed inferior but substantial agreement. All three toxin A/B assays showed almost perfect agreement. Conclusions: Since the QCC GDH and toxin A/B assay is relatively more sensitive and specific than the other two immunoassays for discriminating toxigenic or non-toxigenic CDI, QCC is very helpful for the simultaneous identification of GDH and CD toxin A/B in the initial step of the two-round workflow for diagnosing CDI.

## 1. Introduction

*Clostridioides difficile* (CD), a Gram-positive, anaerobic, spore-forming bacillus, is the major causative agent of antibiotic-induced diarrhea, and toxigenic strains are responsible for *C. difficile* infection (CDI) [1]. However, result discrepancies in diagnostic laboratory testing are an ongoing barrier to public health reporting and medical decision-making [2]. Currently, several methods have been developed based on different assay principles [3,4,5,6] and are available for diagnosing CDI, including the rapid detection of GDH or CD toxin A/B by toxigenic CD culture, tissue culture neutralization (TCN), immunochromatographic test devices, enzyme immunoassay (EIA), and the nucleic acid amplification test (NAAT) for toxin gene detection [7]. Glutamate dehydrogenase (GDH) is a constitutive enzyme that is produced in both non-toxigenic and toxigenic CD strains and related genera similar to CD and other members of the Clostridioides genus [8]. This assay has high negative predictive value and sensitivity, but low specificity [9], whereas the identification of CD toxin A/B in stool samples, although highly specific, has lower sensitivity, leading to a substantial proportion of false negatives [10]. TCN and toxigenic CD culture are regarded as the gold standard for CDI diagnosis [11], but these methods are time-consuming and tedious, requiring laboratory facilities and specific technical expertise [12]. Furthermore, the NAAT for the toxin B gene (tcdB) directly from stool samples has high specificity and sensitivity compared to toxigenic CD culture and TCN. Depending on the NAAT method, it can be expensive, but this approach is usually relatively quick. On the other hand, in the medical laboratory, a step-by-step workflow for CDI detection using GDH and CD toxin A/B assays as screening tests, along with a NAAT, has recently been recommended [13,14]. The VIDAS *C. difficile* GDH and toxin A/B (CDAB) comprises two complementary automated tests based on the enzyme-linked fluorescent assay technique (ELFA) for use on the VIDAS family of instruments (bioMérieux, Marcy-l’Etoile, France). The RIDASCREEN *C. difficile* GDH and toxin A/B (RIDA; R-Biopharm, Darmstadt, Germany) comprises enzyme-linked immunosorbent assay (ELISA) performed using a 96-well microwell plate per batch, independently. C. DIFF QUIK CHEK COMPLETE (QCC; TechLab, Blacksburg, VA, USA), a lateral flow membrane enzyme immunoassay, tests for both GDH and toxin A/B simultaneously in one cartridge. Only comparative evaluations between two immunoassays among the VIDAS, RIDA, and QCC assays for the qualitative detection of CD GDH and CD toxin have been reported [5,6]. In this study, we evaluated these three immunoassays for the simultaneous detection of GDH and CD toxin A/B.

## 2. Materials and Methods

### 2.1. Samples

A total of 304 stool samples submitted to the medical microbiology laboratory at Jeonbuk National University Hospital (Jeonju, Korea) between January 2017 and January 2018 were used. After routine microbiology testing, leftover samples were stored at 4 °C for processing within 24 h and frozen at −70 °C for further evaluation and processing.

### 2.2. Three Immunoassays for the Detection of GDH Antigen and CD Toxin A/B

All stool samples were tested for the presence of GDH antigen and CD toxin A/B via the VIDAS, RIDA, and QCC tests according to the manufacturers’ recommendations.

Briefly, for VIDAS GDH, 200 mg of mixed semi-solid stool or a 200 μL aliquot of well-mixed liquid stool was dispensed into a centrifuge tube. Next, 1000 μL of pretreatment reagent (R1 *C. difficile*) was added to the centrifuge tube, mixed completely, and centrifuged for 10 min at 3000× *g*. A 300 μL quantity of supernatant was collected and added to the sample well of the GDH kit to carry out the assay. Next, stool samples were examined for VIDAS CDAB via the ELFA immunoassay, as previously described [3]. The assay results were regarded as negative, equivocal, or positive according to the fluorescence intensity, as described in the relevant package insert for each assay.

Second, the RIDA GDH and toxin A/B tests were sequentially performed using separate reagents [6]. A total volume of 100 μL of stool sample with biotinylated anti-GDH and CD toxin A/B antibodies was transferred to each sample well and incubated for 60 min at 20–25 °C. After washing with washing buffer 5 times, streptavidin poly-peroxidase conjugates were added and then incubated for 30 min. After washing, the substrates were added, followed by 15 min of incubation and the addition of a stop reagent. The concentrations of GDH and CD toxin A/B were measured at a dual wavelength of 450/630 nm using a GEMINI automated immunoassay system (STRATEC Biomedical, Birkenfeld, Germany).

Third, for QCC, a 500 μL quantity of mixture comprising 25 μL of stool sample with diluent and conjugate (TechLab) was transferred to the device sample well. After incubation for 15 min at 20–25 °C, wash buffer was added, followed by the addition of the substrate (TechLab) to the reaction window. The results were read after 10 min. The presence of GDH antigen and/or CD toxin A/B was indicated by the appearance of a color bar in the appropriate detection zone [6]. The performance characteristic information of the three immunoassays for the simultaneous detection of GDH and CD toxin A/B, as provided by the manufacturers, are summarized in Table 1.

### 2.3. C. difficile Culture and Nucleic Acid Amplification Test for C. difficile Toxin A/B

As a complementary reference method for the GDH antigen results of the three immunoassays, CD culture was performed using ChromID *C. difficile* agar (ChromID CD agar; bioMérieux, Lyon, France) to confirm the presence of CD. Alcohol-shocked stool samples were inoculated on ChromID CD agar and incubated anaerobically at 35 °C for up to 48 h. Gray to black colonies grown on ChromID CD agar were investigated by Gram staining and interpreted using the manufacturer’s guide. When the colony morphology was ambiguous, it was confirmed by VitekMS (BioMérieux, Hazelwood, MI, USA) [15]. As a comparable reference method, the Xpert *C. difficile* assay (Xpert CD assay; Cepheid, Sunnyvale, CA, USA) was performed to confirm the presence of CD toxin A/B using the GeneXpert Dx system according to the manufacturer’s instructions in all CD isolates. The assay uses primers that target the cytotoxin gene (tcdB), binary toxin genes (cdtA and cdtB), and a single-nucleotide deletion at position 117 in the tcdC gene. As a result, the Xpert CD assay can detect toxigenic CD strains and differentiate CD presumptive 027/NAP1/BI.

### 2.4. Statistical Analysis

The sensitivity and specificity of each immunoassay for GDH and toxin A/B were calculated against the results of CD culture and the Xpert *C. difficile* assay as complementary reference methods. In addition, as consensus results were defined as true positive/true negative instead of using reference testing, the estimates were called the positive percentage agreement (PPA) and negative percentage agreement (NPA) rather than sensitivity and specificity. The PPA and NPA are the numbers of positive and negative samples, respectively, from each method among the number of samples showing concordant positive for at least 2 of the 3 assays. Cohen’s kappa was computed to evaluate the inter-rater agreement among the three immunoassays, and the Kappa results can be interpreted as follows: a value of ≤0 indicates no agreement, 0.01–0.20 is none to slight, 0.21–0.40 is fair, 0.41–0.60 is moderate, 0.61–0.80 is substantial, and 0.81–1.00 is almost perfect agreement. The PPA/sensitivity and NPA/specificity of each immunoassay for GDH and toxin A/B were calculated against the consensus results, CD culture, and Xpert *C. difficile* assay using a diagnostic test evaluation calculator provided by MedCalc (https://www.medcalc.org/calc/diagnostic_test.php (accessed on 27 July 2021)). Statistical analyses were performed using MedCalc (version 19.5.3.; MedCalc Software, Ostend, Belgium). A *p*-value of <0.05 by McNemar’s test was considered statistically significant.

## 3. Results

### 3.1. Analytical Performance of the Three GDH Immunoassays as Compared to C. difficile Culture

Among the 304 stool samples, 124 CD isolates were obtained by CD culture. In total, 120, 111, and 118 samples were positive and 131, 138, and 133 were negative for GDH when tested using VIDAS, RIDA, and QCC, respectively. Statistically significant differences between all three GDH immunoassays and CD culture were observed (*p* < 0.0001 for VIDAS GDH and QCC GDH, and 0.0001 for RIDA GDH). All three GDH assays showed overall substantial agreement with the CD culture. The VIDAS GDH (96.8%) and QCC GDH (95.2%) tests showed relatively higher sensitivity of >90% (*p* = 0.0232 and 0.0920, respectively), compared to RIDA GDH (89.5%). The false negative rate of VIDAS GDH (3.2%) was lower than those of the other two GDH immunoassays (RIDA GDH 10.5%, *p* = 0.0232; QCC GDH 4.8%, *p* = 0.5211). However, the specificity did not significantly differ among the three GDH assays (Table 2).

### 3.2. Analytical Performance of the Three Toxin A/B Immunoassays as Compared to the Xpert C. difficile Assay

Of 152 positives for CD toxin A/B obtained by the Xpert *C. difficile* assay, 86, 76, and 78 samples were positive by VIDAS, RIDA, and QCC, respectively, and of 152 negatives, 142, 152, and 148 samples were negative by VIDAS, RIDA, and QCC, respectively. Statistically significant differences between all three toxin A/B immunoassays and the Xpert *C. difficile* assay were observed (*p* < 0.0001 for all three toxin A/B immunoassays). The false negative rates of all three toxin A/B immunoassays were higher than that for the Xpert *C. difficile* assay. All three toxin A/B assays showed overall moderate agreement with the Xpert *C. difficile* assay. VIDAS CDAB and QCC toxin A/B showed relatively higher sensitivity of >50% (*p* = 0.2496 and 0.8210, respectively) but lower specificity of 93.4 and 97.4 (*p* = 0.0013 and 0.0457, respectively), as compared to RIDA toxin A/B (100%). The false positive rate of RIDA toxin A/B (0%) was lower than those of the other two toxin A/B immunoassays (VIDAS CDAB 3.2%, *p* = 0.0264; QCC toxin A/B 2.6%, *p* = 0.0457). However, sensitivity did not significantly differ among the toxin A/B immunoassays (Table 3).

### 3.3. Analytical Performance of the Three GDH Immunoassays as Compared to the Consensus GDH Results

Assay agreements, either positive or negative, for GDH and CD toxin A/B between each immunoassay and the consensus result were estimated. In comparison with consensus GDH results, RIDA GDH and QCC GDH showed no statistically significant difference, with PPAs of 88.2% and 98.7% and NPAs of 92.3% and 95.8%, respectively (*p* = 0.2005 for RIDA GDH and *p* = 0.2891 for QCC GDH). VIDAS GDH and QCC GDH showed almost perfect agreement, whereas RIDA GDH showed inferior but substantial agreement. However, the difference was statistically significant between VIDAS GDH and the consensus result only (*p* = 0.0215). VIDAS GDH (PPA, 99.4%; NPA, 93.7%) and QCC GDH (PPA, 98.7%; NPA, 95.8%) also showed relatively higher PPAs of >90% (*p* < 0.0001 and 0.0001, respectively) and higher NPAs (*p* = 0.6433 and 0.2117, respectively), whereas RIDA GDH showed a relatively lower PPA of 88.2% and a lower NPA of 92.3%. The false negative rate of RIDA GDH (13.4%) was higher than those of the other two toxin A/B immunoassays (VIDAS GDH 0.6%, *p* < 0.0001; QCC GDH 1.2%, *p* < 0.0001) (Table 4).

### 3.4. Analytical Performance of the Three Toxin A/B Immunoassays as Compared to the Consensus Toxin A/B Results

In comparison with the consensus toxin A/B results, QCC toxin A/B showed no statistically significant difference, with a PPA of 91.9% and an NPA of 98.6% (*p* = 0.3438). For CD toxin A/B, similar to the assay agreement for GDH, VIDAS CDAB and QCC toxin A/B showed relatively higher Kappa results of 0.88 and 0.92, respectively, as compared to RIDA toxin A/B with a Kappa value of 0.85, even though all three toxin A/B assays showed almost perfect agreement. VIDAS CDAB (PPA, 96.5%; NPA, 94.0%) and QCC toxin A/B (PPA, 91.9%; NPA, 98.6%) also showed relatively higher PPAs of >90% (*p* = 0.0051 and 0.1014, respectively) and lower or similar NPAs (*p* = 0.0237 and 0.7396, respectively), whereas RIDA toxin A/B showed a lower PPA of 83.7% and an NPA of 98.2%. The false negative rate of VIDAS CDAB (3.5%) was lower than those of the other two toxin A/B immunoassays (RIDA toxin A/B 16.3%, *p* = 0.0051; QCC GDH 8.1%, *p* = 0.1982). However, the false positive rate of VIDAS CDAB (6%) was higher than those of the other two toxin A/B immunoassays (RIDA toxin A/B 1.8%, *p* = 0.0237; QCC toxin A/B 1.4%, *p* = 0.0110) (Table 4).

## 4. Discussion

As the incidence and severity of CDI increase every year [16], accurate, readily available, and rapid detection has become extremely important. Even though CDI patients in Asia-Pacific countries exhibit the typical characteristics of CDI seen elsewhere, the outcomes appear to be less severe in Asia-Pacific countries [17]. Simultaneous testing for both GDH and CD toxin A/B has the benefit of being able to accurately and rapidly distinguish whether CDI produces toxins or not as an initial test and is relatively less labor-intensive and time-consuming than conventional toxigenic CD culture [3,4,5,6]. Several studies have evaluated the analytical performance of each of the EIA [3,4,5,6] or NAAT [18,19,20,21] methods, as compared to a reference method. Conventional EIA for CD toxin A/B detection is currently the most commonly used method for CDI diagnosis, but its low sensitivity necessitates the development of an alternative strategy to improve the diagnosis in developing countries [22]. The NAAT provides high sensitivity in CDI diagnosis, but it may affect the ability to distinguish between low-level colonization and true CDI [23]. This emphasizes a careful interpretation in conjunction with clinical evaluation and the requirement for strict pre-analytical inclusion/exclusion criteria of submitted samples [24]. Furthermore, it has been proposed that the NAAT for detecting CD may be less specific than EIA assays [19]. Since several immunoassays that can detect the presence of CD and/or CD toxin A/B have been developed, the application of a step-by-step workflow for CDI diagnosis is recommended in medical microbiology laboratories [13,25].

In this study, the analytical performance of three immunoassays widely used for GDH and CD toxin A/B detection were evaluated in comparison to CD culture using ChromID agar and the Xpert *C. difficile* assay as a presumptive reference method. The three GDH immunoassays showed satisfactory analytical sensitivities from 89.5% to 96.8%; however, they showed lower analytical specificities from 72.8% to 76.7%, in the range of previous reports (sensitivities, 81.0–100%; specificities, 82.0–94.8%) [5,6,26,27], when compared to CD culture. With a range similar to those in previous studies (sensitivities, 48–67%; specificities, 94.4–100%) [5,6,26,27], the three CD toxin A/B immunoassays showed low analytical sensitivities, from 50.0% to 56.6%, and analytical specificities from 93.4% to 100%, when compared to the Xpert *C. difficile* assay. In the comparison of the consensus GDH results and the CD culture results, a high false negative rate in the CD culture was suspected. In particular, 36 inconclusive results of negatives for CD culture and positives for the Xpert *C. difficile* assay were identified, which paradoxically means that CD toxin A/B was present in the absence of *C. difficile* isolates. In prior studies, prolonging incubation on ChromID *C. difficile* agar for 48 h in cases of negative results at 24 h enhanced the recovery of CD strains from 74.1% to 87% [28] and from 58.3% to 100% [29]. However, extending incubation may increase indirect costs and the turnaround time. When referring to the consensus results, Cases A to D (N = 9) are likely to be toxigenic CDI because of the positives for GDH in all three GDH immunoassays, in addition to the positives for CD toxin A/B in the Xpert *C. difficile* assay and at least two of the three CD toxin A/B immunoassays, even though the CD culture was suspected of being a false negative. Similarly, Cases E to G (N = 21) are likely to be non-toxigenic CDI because of the positives for GDH in at least two of the three GDH immunoassays, in addition to the positives for CD toxin A/B in the Xpert *C. difficile* assay and at most one of the three CD toxin A/B immunoassays, even though the CD culture was suspected of being a false negative. However, the Xpert *C. difficile* assay was suspected of returning a false positive in Cases H and I (N = 6) and a false negative in Case J (N = 3), referring to the consensus results (Table 5). Interestingly, one sample (sample #214) was positive for GDH and CD toxin A/B by both VIDAS and QCC, but was determined to be negative by toxigenic CD culture and the Xpert *C. difficile* assay. Thus, it was classified as a false positive result for toxigenic CDI. Cross-reactivity may be explained as the cause of this discrepancy, because toxins formed by other clostridial species, such as *C. sordellii*, are antigenically similar to those formed by *C. difficile* [30].

**Figure 1:**
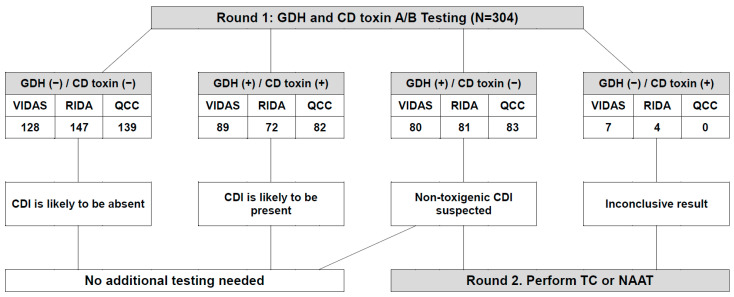
Two-round workflow for the diagnosis of toxigenic *Clostridioides difficile* infection by applying GDH and CD toxin A/B testing. Abbreviations: GDH, glutamate dehydrogenase; CD, *Clostridioides difficile*; (−), negative; (+), positive; VIDAS, VIDAS *C. difficile* GDH and toxin A&B; RIDA, RIDASCREEN *C. difficile* GDH and toxin A/B; QCC, C. DIFF QUIK CHEK COMPLETE; CDI, *Clostridioides difficile* infection; TC, toxigenic *Clostridioides difficile* culture; NAAT, nucleic acid amplification test.

In the two-round workflow for the diagnosis of CDI by applying GDH and CD toxin A/B testing, when GDH and CD toxin A/B were both negative or both positive, the use of VIDA, RIDA, and QCC for first-round testing in a two-round workflow eliminated the requirement for second-round testing in 71.4%, 72.0%, and 72.7% of the stool samples, respectively. When non-toxigenic CDI or a false negative for toxigenic CDI was suspected, after the use of VIDA, RIDA, and QCC, subsequent toxigenic CD culture or a toxin gene NAAT as second-round testing may be needed in 26.3%, 26.6%, and 27.3% of the stool samples, respectively. Interestingly, QCC showed no inconclusive results; however, VIDAS and RIDA showed seven and four inconclusive results, respectively (Figure 1). Among the inconclusive results, of the seven (2.3%) by VIDAS, six were negative, but only one (sample #286) was positive in the CD culture and Xpert *C. difficile* assay. Comparing these to the other two immunoassays, six were negative and only one (sample #11) was positive for QCC GDH and toxin A/B. Of the four (1.3%) inconclusive results by RIDA, all four were positive in the CD culture and Xpert *C. difficile* assay. Comparing these to the other two immunoassays, four in VIDAS GDH, three in VIDAS CDAB, two in QCC GDH, and two in QCC toxin A/B were positive. Thus, no additional testing was required when QCC was used as an initial screening test, if GDH positivity and CD toxin A/B negativity are regarded as indicating non-toxigenic CDI only. Alternatively, to estimate the possibility of determining the existence of *C. difficile* and toxigenic CDI using the Xpert *C. difficile* assay, RIDA GDH showed no statistically significant difference, with the best sensitivity of 91.5% and specificity of 90.8%, in comparison between the Xpert *C. difficile* assay and the three GDH immunoassays (*p* = 1.000). Thus, RIDA GDH and toxin A/B may be the best candidate if only one testing workflow for CDI diagnosis should be applied when NAAT or toxigenic CD culture are not available. Overall, of the three immunoassays, QCC GDH and toxin A/B showed no significant difference when compared to the consensus GDH and toxin A/B results. In particular, QCC based on this simple and rapid test device has the advantage of simultaneous detection of GDH and CD toxin A/B, which can be individually tested at the same time in a single reaction well, without needing to be batched. Furthermore, no special hardware equipment for test processing and detection is required to use this device, and it can be easily implemented in medical microbiology laboratories. However, VIDAS and RIDA are suitable for high-throughput batch testing in medical microbiology laboratories that require analyses of large numbers of stool samples. The different requirements in platform characteristics, testing capacity, repeat testing rates, and turnaround times could help medical microbiology laboratories decide which assay would integrate better into their setting and to better select an immunoassay platform for CDI diagnosis.

## 5. Conclusions

In conclusion, all three immunoassays had similar high sensitivity for GDH and low specificity for CD toxin A/B when compared to CD culture and the Xpert *C. difficile* assay, respectively. Furthermore, the PPAs and NPAs between all three GDH and toxin A/B immunoassays were highly concordant, based on consensus results. Since QCC GDH and toxin A/B is relatively more sensitive and specific than the other two immunoassays for discriminating toxigenic or non-toxigenic CDI, QCC is very helpful for the simultaneous identification of GDH and CD toxin A/B in the initial step of a two-round workflow for diagnosing CDI.

## Figures and Tables

**Table 1:** Performance characteristic information of the three immunoassays for the detection of *Clostridium difficile* glutamate dehydrogenase and toxin A/B, provided by the manufacturers.

Characteristic	VIDAS *C. difficile* GDH and Toxin A&B	RIDASCREEN *C. difficile* GDH and Toxin A/B	C. DIFF QUIK CHEK COMPLETE
Manufacturer	bioMérieux	R-Biopharm	TechLab
Immunoassay system	VIDAS	GEMINI	Not required
Principle of operation	Enzyme-linked fluorescent assay	Enzyme-linked immunosorbent assay	Lateral flow membrane enzyme assay
Testing concurrency *	Separately	Separately	Simultaneously
Sample type	Stool	Stool	Stool
Minimum sample volume	200 μL	100 μL	25 μL
Running time of process	90 min	120 min	30 min

* Whether GDH and toxin A/B are detected separately or simultaneously when testing.

**Table 2:** Analytical performance results among the three immunoassays for the detection of *Clostridioides difficile* glutamate dehydrogenase, as compared with *Clostridioides difficile* culture.

Immunoassay	Result	*C. difficile* Culture		Kappa (95% CI)	Sensitivity, % (95% CI)	Specificity, % (95% CI)	*p*-Value
		Positive (*n* = 124)	Negative (*n* = 180)				%Diff (95% CI)
VIDAS GDH	Positive	120	49	0.66 (0.58–0.74)	96.8 (92.0–99.1)	72.8 (65.7–79.1)	<0.0001
	Negative	4	131				−14.52 (−18.89 to −10.15)
RIDA GDH	Positive	111	42	0.64 (0.55–0.72)	89.5 (82.7–94.3)	76.7 (69.8–82.6)	0.0001
	Negative	13	138				−9.54 (−14.2 to −4.88)
QCC GDH	Positive	118	47	0.66 (0.58–0.74)	95.2 (89.8–98.2)	73.9 (66.8–80.1)	<0.0001
	Negative	6	133				−13.49 (−17.93 to −9.04)

**Table 3:** Analytical performance results among the three immunoassays for the detection of *Clostridioides difficile* glutamate dehydrogenase and toxin A/B, as compared with the Xpert *C. difficile* assay.

Immunoassay	Result	Xpert *C. difficile* Assay		Kappa (95% CI)	Sensitivity, % (95% CI)	Specificity, % (95% CI)	*p*-Value
		GDH					%Diff (95% CI)
		Positive (*n* = 152)	Negative (*n* = 152)				
VIDAS GDH	Positive	143	26	0.77 (0.70–0.84)	94.1 (89.1–97.3)	82.9 (76.0–88.5)	0.0060
	Negative	9	126				−5.59 (−9.35 to −1.83)
RIDA GDH	Positive	139	14	0.82 (0.76–0.87)	91.5 (85.8–95.4)	90.8 (85.0–94.9)	1.0000
	Negative	13	138				−0.33 (−3.68 to 3.02)
QCC GDH	Positive	141	24	0.77 (0.70–0.84)	92.8 (87.4–96.3)	84.2 (77.4–89.6)	0.0410
	Negative	11	128				−4.28 (−8.06 to −0.49)
		**CD toxin A/B**					
		**Positive (*n* = 152)**	**Negative (*n* = 152)**				
VIDAS CDAB	Positive	86	10	0.5 (0.41–0.59)	56.6 (48.3–64.6)	93.4 (88.2–96.8)	<0.0001
	Negative	66	142				18.42 (13.2 to 23.65)
RIDA toxin A/B	Positive	76	0	0.5 (0.42–0.58)	50.0 (41.8–58.2)	100 (97.6–100)	<0.0001
	Negative	76	152				25 (20.13 to 29.87)
QCC toxin A/B	Positive	78	4	0.49 (0.40–0.57)	51.3 (43.1–59.5)	97.4 (93.4–99.3)	<0.0001
	Negative	74	148				23.03 (17.95 to 28.1)

GDH, glutamate dehydrogenase; CD toxin A/B, *C. difficile* toxin A/B.

**Table 4:** Consensus results of assay agreement among the three immunoassays for the detection of *Clostridioides difficile* glutamate dehydrogenase and toxin A/B.

Immunoassay	Result	Consensus Result		Kappa (95% CI)	PPA, % (95% CI)	NPA, % (95% CI)	*p*-Value
		GDH					%Diff (95% CI)
		Positive (*n* = 161)	Negative (*n* = 143)				
VIDAS GDH	Positive	160	9	0.94 (0.89–0.97)	99.4 (96.6–100)	93.7 (88.4–97.1)	0.0215
	Negative	1	134				−2.63 (−4.65 to −0.61)
RIDA GDH	Positive	142	11	0.80 (0.94–0.87)	88.2 (82.2–92.7)	92.3 (86.7–96.1)	0.2005
	Negative	19	132				2.63 (0.89 to 6.12)
QCC GDH	Positive	159	6	0.95 (0.91–0.98)	98.7 (95.6–100)	95.8 (91.1–98.4)	0.2891
	Negative	2	137				−1.32 (−3.13 to 0.5)
		**CD toxin A/B**					
		**Positive** **(*n* = 86)**	**Negative** **(*n* = 218)**				
VIDAS CDAB	Positive	83	13	0.88 (0.82–0.94)	96.5 (90.1–99.3)	94.0 (90.0–96.8)	0.0213
	Negative	3	205				−3.29 (−5.84 to −0.74)
RIDA toxin A/B	Positive	72	4	0.85 (0.78–0.92)	83.7 (74.2–90.8)	98.2 (95.4–99.5)	0.0309
	Negative	14	214				3.29 (0.58 to 6)
QCC toxin A/B	Positive	79	3	0.92 (0.87–0.97)	91.9 (84.0–96.7)	98.6 (96.0–99.7)	0.3438
	Negative	7	215				1.32 (−0.72 to 3.35)

PPA, positive percentage agreement; NPA, negative percentage agreement; GDH, glutamate dehydrogenase; CD toxin A/B, *C. difficile* toxin A/B.

**Table 5:** Suspected inconclusive results interpreted by *C. difficile* culture and the Xpert *C. difficile* assay as compared to the three GDH and CD toxin A/B immunoassays.

Case (N = 39)	CD Culture	GDH			Xpert Assay	CD Toxin A/B		
		VIDAS	RIDA	QCC		VIDAS	RIDA	QCC
A (N = 5)	Neg	Pos	Pos	Pos	Pos	Pos	Pos	Pos
B (N = 1)	Neg	Pos	Pos	Pos	Pos	Pos	Pos	Neg
C (N = 2)	Neg	Pos	Pos	Pos	Pos	Pos	Neg	Pos
D (N = 1)	Neg	Pos	Pos	Pos	Pos	Neg	Pos	Pos
E (N = 1)	Neg	Pos	Pos	Pos	Pos	Neg	Pos	Neg
F (N = 17)	Neg	Pos	Pos	Pos	Pos	Neg	Neg	Neg
G (N = 3)	Neg	Pos	Neg	Pos	Pos	Neg	Neg	Neg
H (N = 5)	Neg	Neg	Pos	Neg	Pos	Neg	Neg	Neg
I (N = 1)	Neg	Neg	Neg	Neg	Pos	Neg	Neg	Neg
J (N = 3)	Neg	Pos	Pos	Pos	Neg	Neg	Neg	Neg

CD Culture, *C. difficile* culture using ChromID *C. difficile* agar; GDH, glutamate dehydrogenase; Xpert assay, Xpert *C. difficile* assay; CD toxin A/B, *C. difficile* toxin A/B; Neg, negative; Pos, positive.

## Data Availability

Not applicable.
